# Validation of Serbian Version of Dysfunctional Voiding Symptom Score (DVSS) Questionnaire

**DOI:** 10.3390/jcm7080217

**Published:** 2018-08-14

**Authors:** Dragana Cirovic, Ivana Petronic, Dejan Nikolic, Tatjana Knezevic, Vojkan Vukadinovic, Polina Pavicevic

**Affiliations:** 1 Faculty of Medicine, University of Belgrade, 11000 Belgrade, Serbia; ivana.pm@live.com (I.P.); denikol27@gmail.com (D.N.); vojkan.vukadinovic@udk.bg.ac.rs (V.V.); pzmbov@yahoo.com (P.P.); 2 Physical Medicine and Rehabilitation Department, University Children’s Hospital, Tirsova 10, 11000 Belgrade, Serbia; tknezevic@gmail.com; 3 Pediatric Surgery Department, University Children’s Hospital, 11000 Belgrade, Serbia; 4 Pediatric Radiology Department, University Children’s Hospital, 11000 Belgrade, Serbia

**Keywords:** validation, questionnaire, dysfunctional voiding, children

## Abstract

Objective: The aims of our study were to translate the dysfunctional voiding symptom score (DVSS) from English to Serbian; culturally adopt the items; assess the internal consistency and the test–retest reliability of DVSS_SR_ in patients with dysfunctional voiding (DV); evaluate and test the construct and divergent validity of DVSS_SR_ against demographic parameters (gender and education); and examine the level of explained variability for each item of DVSS_SR_ against demographic parameters (gender and education). Methods: The cross-sectional observational study included 50 patients with dysfunctional voiding aged 5 years and above. The DVSS questionnaire was translated from English into Serbian by the forward–backward method. Internal consistency was assessed with Cronbach α and test–retest reliability with intraclass correlation coefficient (ICC). For validity testing we performed construct and divergent validity analyses. Results: There was excellent internal consistency for every item except for Item 6 (0.787) and Item 3 (0.864), where internal consistency was good. The observed test/retest ICC for average measures was more than 0.75 (excellent) for all DVSS_SR_ items. Gender and educational level does not correlate significantly with each item of DVSS_SR_ (*p* > 0.05). For divergent validity, there were no significant differences in mean values of each item of DVSS_SR_ between genders and different levels of education (*p* > 0.05). Variability that can be explained for gender and educational level was below 10%. Conclusion: Translated DVSS_SR_ is of adequate validity and reliability for assessing DV in children.

## 1. Introduction

Dysfunctional voiding (DV) is a frequent clinical condition in pediatric urology, with female predominance (5:1 female-to-male ratio), and is approximately seen in 40% of patients [1,2]. Such a condition can be defined as “habitual contraction of the urethral sphincter during voiding” [3]. Patients with DV usually present with recurrent urinary tract infections, along with urinary incontinence and bowel problems (constipation and encopresis) [4]. In order to evaluate DV and improve diagnostics of such condition, Farhat et al. developed a questionnaire that assesses symptom scoring for wetting and functional disorders in children [5]. This questionnaire was named the DV Symptom Score (DVSS). So far, the DVSS was used in many studies for the follow-up of patients with DV, and for the estimation of treatment effectiveness [6,7,8]. It was also used in DV patients with attention-deficit or hyperactivity disorder [6]. Considering the diagnosis of DV, it was stated that the specificity of DVSS was 97.6% and its accuracy was 76% [6]. To the best of our knowledge, so far DVSS has been validated in several countries [4,9,10]. Since in Serbia there is no questionnaire that is designed to evaluate DV, validation of DVSS will be of significant benefit in assessment of DV in pediatric population. Since the DVSS was designed in English, cultural adaptation during the validation process of the Serbian version of DVSS is needed.

Therefore, the first aim of our study was to translate DVSS from English to Serbian and to culturally adopt the items of the questionnaire due to the possible cultural differences among the two populations. The second aim was to assess the internal consistency and the test/retest reliability of DVSS_SR_ in patients with DV. The third aim was to evaluate and test the construct and divergent validity of DVSS_SR_ against demographic parameters (gender and education). The fourth aim was to examine the level of explained variability for each Item of DVSS_SR_ against demographic parameters (gender and education). 

## 2. Material and Methods

### 2.1. Study Population

The cross-sectional observational study included 50 patients with DV that were treated at University Children's Hospital in Belgrade, Serbia. The participants were invited into the study by telephone or email contact from archives of medical documentation between August 2017 and September 2017. The eligibility criteria were a DV diagnosis of more than a year ago, absence of structural causes of DV, and an age of 5 years and above. Confirmation of DV was done by repeated uroflow measurements, along with electromyography (EMG) with surface electrodes (3 measurements were done on separate occasions and mean value was calculated) by a Board-certified urologist. Patients were tested on 2 occasions: test and retest (after 3 days). The parents or legal guardians of eligible patients were informed about the study protocol and consent was obtained. The study was approved by Institutional Review Board and followed the principles of good clinical practice. 

### 2.2. DVSS Questionnaire

The DVSS questionnaire was translated from English into Serbian and validated after permission for translation by one of the DVSS authors, Prof. Walid Farhat. The DVSS questionnaire consists of 10 questions, of which 9 are graded with scores from 0–3 (0—almost never, 1—less than half the time, 2—about half the time, 3—almost every time), and with an additional option of “Not Available”, while the 10th question is graded with two scores: NO (0) and YES (3) [5]. This questionnaire is used for the scoring of wetting and functional disorders in children; thus, it is useful in pediatric populations for the objective grading of DV.

### 2.3. Translation and Validation Process

The DVSS questionnaire was translated from English into Serbian by the forward–backward method [11,12] and following the principles of framework for translation and cultural adaptation of patient-reported outcome measures [13]. The translation of the DVSS questionnaire into Serbian was done independently by two lead authors who knew the study objectives. The first author, who acquired written permission for translation by e-mail, was in contact with the original author of the questionnaire. In a forward translation, two versions (DVSS 1a and DVSS 1b) were generated. In a reconciliation step, these two versions were compared, analyzed, and merged into a final forward translation (DVSS 1.0) under the supervision of an independent translator who was unaware of the study objective, who lived for more than a year in an English-speaking country, and is fluent in English and Serbian. The further step included a back-translation into English by another independent translator who was fluent in English and Serbian and unaware of study objectives. The differences between the forward and the backward translations were discussed and settled through consensus. Since there were no major linguistic discordant, neither independent expert panel nor the original DVSS developers were invited in this stage of the validation process. The final version of the DVSS (DVSS 2.0) questionnaire was initially presented to 7 patients who were not included into the study for screening of question-item appropriateness and linguistic understanding. Parents or legal guardians were instructed to provide feedback in a case where questions were not well understood. We received no feedback concerning difficulties in item interpretation and answering during the debriefing step, and the expert panel that consisted of 2 independent pediatric urologists and 1 independent physiatrist with subspecialist interests in voiding dysfunctions approved the Serbian version of DVSS 3.0 as final (DVSS_SR_).

## 3. Statistical Analysis

### 3.1. Internal Consistency and Reliability Testing

The internal consistency of the evaluated items of the DVSS_SR_ questionnaire was analyzed using the Cronbach α on both occasions (test and retest). The favorable value of Cronbach α, which indicates satisfactory internal consistency, was set at >0.70; categories were: >0.9 was considered excellent, 0.8–0.9 good, 0.7–0.8 acceptable, 0.6–0.7 questionable, and 0.5–0.6 poor internal consistence [14]. Test–retest reliability was examined with the intraclass correlation coefficient (ICC). The values >0.90 in ICC were considered as evidence of stability [4]. However, the single-measure ICC was reported and interpreted as poor (<0.40), moderate (0.40–0.59), good (0.60–0.74), and excellent (≥0.75) [15].

### 3.2. Validity Testing

For construct validity, every item (Items 1–10) of DVSS_SR_ was correlated with gender (1–2 each, where 1—males and 2—females) and educational level (1–2 each, where 1—preschool; 2—primary school). To assess the strength of association, we applied further interpretation of results: very large (*r* > 0.7), large (0.50 < *r* < 0.70), medium (0.30 < *r* < 0.50), and small (0.10 < *r* < 0.30) [16]. For evaluation of divergent validity, the study sample was split by gender (male versus female) and educational level (preschool versus primary school) and the DVSS_SR_ scores were compared using the unpaired *t*-test.

### 3.3. Explained Variability Testing

To explain and quantify variability that can be explained between different degrees for each Item for DVSS_SR_ and Gender or Education, we introduced η2 = Sum of squares (Between groups)/Sum of squares (Total) × 100, where sum of squares was generated from a one-way ANOVA test and results were presented as percentage (%) [17].

## 4. Results

In Table 1, we presented demographic characteristics of evaluated population. Mean values of DVSS were presented for every item and as total score on both occasions (Test and Retest). There were no significant differences in values of obtained scores between Test and Retest (*p* > 0.05) (Table 1).

Regarding the DVSS questionnaire, there was excellent internal consistency for every item except for Item 6 (0.787) and Item 3 (0.864), where internal consistency was good (Table 2). Pearson’s correlation that was used to assess reliability showed significant positive correlation between Test and Retest (Table 2), with highest correlation for Item 7 (*r* = 0.964), and lowest correlation for Item 3 (*r* = 0.824). 

The observed test/retest ICC for single measures was more than 0.75 (excellent) for all DVSS_SR_ items except for Item 6 (0.652), where such association was good (Table 3). The observed test/retest ICC for average measures was more than 0.75 (excellent) for all DVSS_SR_ items (Table 3). For single measures, the lower limit of 95% confidence interval was marginally lower than 0.75 for Item 2 (0.742), while for Item 3 it was in “good” territory (0.614), and Item 6 was in “moderate” territory (0.458) (Table 3). For average measures, the lower limit of 95% confidence interval was above 0.75 for all items except for Item 6, which was in “good” territory (0.628) (Table 3).

Gender does not correlate significantly with each item of DVSS_SR_ (*p* > 0.05). Same applies for educational level (*p* > 0.05) (Supplementary Material Table S1). 

Regarding divergent validity, there were no significant differences in mean values of each item of DVSS_SR_ between genders (*p* > 0.05). Same applies for educational level (*p* > 0.05) (Supplementary Material Table S2).

For each item of DVSS_SR_, variability that can be explained for gender was below 10%, with the highest for Item 9 (7.05%) (Supplementary Material Table S3). Variability of items of DVSS_SR_ that can be explained by educational level was also below 10%, with the highest for Item 4 (5.69%) (Supplementary Material Table S3).

## 5. Discussion

During the translation process, we followed the principles of conceptual equivalence in order to achieve conceptual similarity of a different culture on proposed and implemented grammatical changes. Since transcultural adaptations are very delicate during the validation process of standardized questionnaires, in order to preserve items' sensitivity, it is important to stress out that there is still no clear consensus regarding the best strategy for such action. Thus, we followed the principles of good practice for the translation and cultural adaptation process for patient-reported outcomes measures [13].

Over the period of translation and feedback of the approved DVSS_SR_ instrument to selected group of patients with DV during the validation process, we did not observed difficulties in translation nor misinterpretation during the feedback process. Thus, despite the necessity for minor cultural adaptations, items of DVSS_SR_ address the same meaning of the original version of DVSS that was in the English language. As it was noticed previously for the Korean and Chinese versions of DVSS, particularly Item 3 for the Korean and Item 5 for the Chinese version, confusions were observed, and thus such items were modified [18]. Considering Item 5, instead of “I only go to the bathroom one or two times each day”, we translated it to “I only go to urinate one or two times each day”. During the reconciliation step and back-translation step, since items were not complex, were easily understood, and the descriptive terms were common to similar cultures, we engaged a translator without prior experience in patient-reported outcomes. Since we haven’t been asked for additional adjustments of items and a better explanation for them during the feedback process, we have demonstrated successful linguistic translation and cultural adaptation of DVSS_SR._

Considering the most appropriate option to measure and express internal consistency, it should be stressed that so far there has been no complete definition of such a term. Thus, several interpretations and definitions were proposed [19,20,21]. Given the fact that, for our validation scale, we have had no subscales or subtests, the DVSS_SR_ questionnaire might not be considered as potentially factorially homogenous; therefore, a model based on average interitem correlations rather than general factor saturation, in our case, should be the advantageous choice for internal consistency evaluation. It should be mentioned that, with regards to the general factor-saturation model, there are several methods in assessing internal consistency (alpha, beta, and hierarchical coefficient omega) [22,23]. Since the adopted and translated version of DVSS_SR_ scale does not contain subscales and subtests and, thus, might be considered not multidimensional, we have agreed to proceed with an internal consistency evaluation by the proposed method of Cronbach [20]. Despite the fact that an average interitem correlation method is not advisable for evaluation of variability among interitem correlations, our decision for the Cronbach method was justified by the fact that we gained no extreme correlation values. Further, in the study of Tang et al. [24], it was pointed out that alpha tends to be most influenced by the test length among the other two indices (beta and hierarchical coefficient omega). However, during the validation process of DVSS_SR_, we didn't modify the number of questions from the original DVSS, and retesting of DVSS_SR_ had the same number of questions as there were during the testing session; thus, the influence of length was assumed to be null in this case.

In our study, we have demonstrated satisfactory internal consistency that was expressed as values of Cronbach alpha of DVSS_SR_ evaluated items with the highest consistency for item 7 and lowest for item 6. Previous studies have demonstrated similar satisfactory consistency for items of DVSS [4,9]. However, it might be assumed to a certain degree that, in the Korean version of DVSS, slightly higher values of Cronbach alpha than ours were due to the possible cultural differences that are particularly expressed in younger age. In the study of Farver et al., it was stressed that cultural individual differences exist between Korean-American and European-American preschoolers [25]. Such differences include: nature of activities, cultural goals, values and beliefs, as well as social interaction and self-expression [25]. Since, the Serbian population regarding cultural patterns is considered to be similar with European populations, the above mentioned cultural differences are thought to be potential factors that might explain, to a certain degree, slight discrepancies in values of Cronbach alpha. Children in Serbia, due to these cultural patterns, to a certain degree, might feel discomfort when performing a “pee dance” or “leg crossing”, since such actions are publicly visible. Therefore, it could have potential influence on Item 6.

The necessity for evaluation of reliability, particularly in the validation process of certain scales and questionnaires, is based on the assumption that the test, which has high reliability, does not need to have high internal consistency [24]. One might not misinterpret the terms’ reliability where data from repeated tests are needed (retest) with internal consistency reliability where estimation is based on single-test data. It should also be underlined that reliability depends on the test length, while such a parameter should be independent for internal consistency evaluation. Therefore, reliability could be increased by increase of test length without conditional changes in internal consistency.

Our study demonstrated excellent test/retest reliability for single measures except for Item 6 of DVSS_SR_, where test/retest reliability was good. Such lower reliability might be explained to a certain degree that the information contained in Item 6 could indicate the impact of dysfunctional voiding in a unique way. However, when reliability was tested for average measure, we gained excellent test/retest reliability.

Considering the necessity for implementation of construct validity in validation of the DVSS_SR_ questionnaire, it should be stated that for such type of validity, Messick identified six contributors: content relevance and technical quality; theoretical understanding of scores and associated empirical evidence, including process analyses; structural data; generalizability; external correlates; and consequences of score interpretation [26,27]. Bearing in mind construct homogeneity, we have assumed that neither gender nor educational level should be of influence with regards to the items in the DVSS_SR_ questionnaire. It was shown from the results of our study that there were no significant correlations between DVSS_SR_ and gender and between DVSS_SR_ and education level.

Despite the fact that we have used divergent validity in our study, it was proposed that such a term preferably should be discriminant validity, since the term “discriminant” more closely reflects the test's ability to discriminate between two or more groups. Since we found no significant differences for both gender and education level when individuals were tested for DVSS_SR_, we pointed out to the evidence of divergent validity of DVSS_SR,_ since observed variables (gender and educational level) are unlikely to explain the influence of item-content understanding on DVSS_SR-_gained values. Further, this was confirmed in our study by explained variability of each item with regards to gender and educational level. 

It should be underlined that there are several limitations to this study. Even though the present study adhered to recommended standards for translation of patient-reported outcomes in a significant manner [13], it did not include a professional translator or an expert panel. The small sample size of the study was the limiting factor disabling the performance of exploratory factor analysis; thus, further study is advised on a larger sample of participants.

## 6. Conclusions

In conclusion, the translated DVSS_SR_ is of adequate validity and reliability for assessing DV in children.

## Figures and Tables

**Table 1:** Demographics and mean values of Dysfunctional Voiding Symptom Score.

Demographics		Values		
**Gender**	Male *N* (%)	25 (50%)		
	Female *N* (%)	25 (50%)		
Age mean ± SD		7.18 ± 1.59		
**Educational level**	Preschool *N* (%)	20 (40%)		
	Primary school *N* (%)	30 (60%)		
Test items		Test (mean ± SD)	Retest (mean ± SD)	*p* value *
Item 1		1.76 ± 0.74	1.68 ± 0.74	0.603
Item 2		1.90 ± 0.84	1.78 ± 0.89	0.562
Item 3		1.46 ± 0.84	1.38 ± 0.81	0.697
Item 4		1.88 ± 0.92	1.90 ± 0.91	0.912
Item 5		1.58 ± 0.64	1.64 ± 0.63	0.749
Item 6		1.30 ± 0.58	1.28 ± 0.73	0.897
Item 7		2.16 ± 0.71	2.12 ± 0.75	0.826
Item 8		1.58 ± 0.91	1.62 ± 0.90	0.865
Item 9		1.88 ± 0.66	1.92 ± 0.63	0.772
Item 10		1.44 ± 1.51	1.38 ± 1.51	0.865
Total score		16.90 ± 4.41	16.64 ± 4.70	0.904

* Mann–Whitney U test.

**Table 2:** Test/retest internal consistency (Cronbach α) and correlation analysis of the Serbian version of the Dysfunctional Voiding Symptom Score.

Test/Retest Items	Cronbach α	Pearson’s Coefficient	
		*r* Values	*p* Values
Item 1	0.922	0.858	0.000
Item 2	0.919	0.847	0.000
Item 3	0.864	0.824	0.000
Item 4	0.930	0.866	0.000
Item 5	0.906	0.828	0.000
Item 6	0.787	0.665	0.000
Item 7	0.981	0.964	0.000
Item 8	0.922	0.901	0.000
Item 9	0.979	0.954	0.000
Item 10	0.934	0.880	0.000

**Table 3:** Test/retest reliability (interclass correlation coefficient) of the Serbian version of the Dysfunctional Voiding Symptom Score.

Test/Retest Items	Intraclass Correlation Coefficient (ICC)-Single Measures		ICC-Average Measures	
	Values	95% CI	Values	95% CI
Item 1	0.853	0.755–0.914	0.921	0.860–0.955
Item 2	0.846	0.742–0.910	0.916	0.852–0.953
Item 3	0.760	0.614–0.856	0.864	0.761–0.922
Item 4	0.872	0.784–0.925	0.931	0.879–0.961
Item 5	0.827	0.716–0.898	0.906	0.834–0.946
Item 6	0.652	0.458–0.787	0.790	0.628–0.881
Item 7	0.962	0.935–0.978	0.981	0.966–0.989
Item 8	0.855	0.758–0.915	0.922	0.862–0.955
Item 9	0.957	0.926–0.976	0.978	0.962–0.988
Item 10	0.878	0.794–0.929	0.935	0.885–0.963

## Supplementary Materials

The following are available online at http://www.mdpi.com/2077-0383/7/8/217/s1, Table S1: Construct validity of the Serbian version of the Dysfunctional Voiding Symptom Score, Table S2. Divergent validity of the Serbian version of the Dysfunctional Voiding Symptom Score, Table S3. Explained variability between gender or and education for each item of the Serbian version of the Dysfunctional Voiding Symptom Score.

## References

[1] Feldman A.S., Bauer S.B. (2006). Diagnosis and management of dysfunctional voiding. Curr. Opin. Pediatr..

[2] Swithinbank L.V., Carr J.C., Abrams P.H. (1994). Longitudinal study of urinary symptoms in children. Longitudinal study of urinary symptoms and incontinence in local schoolchildren. Scand. J. Urol. Nephrol. Suppl..

[3] Arlen A.M. (2017). Dysfunctional Voiders-Medication versus Urotherapy?. Curr. Urol. Rep..

[4] Lee H.E., Farhat W., Park K. (2014). Translation and linguistic validation of the Korean version of the dysfunctional voiding symptom score. J. Korean Med. Sci..

[5] Farhat W., Bägli D.J., Capolicchio G., O’Reilly S., Merguerian P.A., Khoury A., McLorie G.A. (2000). The dysfunctional voiding scoring system: Quantitative standardization of dysfunctional voiding symptoms in children. J. Urol..

[6] Altan M., Çitamak B., Bozaci A.C., Mammadov E., Doğan H.S., Tekgül S. (2017). Is There Any Difference Between Questionnaires on Pediatric Lower Urinary Tract Dysfunction?. Urology.

[7] Alyami F., Ewida T., Alhazmi H., Trbay M., Arafa M., Tahir M., Neel K.F. (2018). Biofeedback as single first-line treatment for non-neuropathic dysfunctional voiding children with diurnal enuresis. Can. Urol. Assoc. J..

[8] Chang S.J., Yang S.S. (2018). Do uroflowmetry and post—Void residual urine tests necessary in children with primary nocturnal enuresis?. Int. Braz. J. Urol..

[9] Calado A.A., Araujo E.M., Barroso U., Netto J.M., Filho M.Z., Macedo A., Bagli D., Farhat W. (2010). Cross-cultural adaptation of the dysfunctional voiding score symptom (DVSS) questionnaire for Brazilian children. Int. Braz. J. Urol..

[10] Chang S.J., Chen T.H., Su C.C., Yang S.S. (2012). Exploratory factory analysis and predicted probabilities of a Chinese version of Dysfunctional Voiding Symptom Score (DVSS) questionnaire. Neurourol. Urodyn..

[11] Bullinger M., Alonso J., Apolone G., Leplège A., Sullivan M., Wood-Dauphinee S., Gandek B., Wagner A., Aaronson N., Bech P. (1998). Translating health status questionnaires and evaluating their quality: The IQOLA Project approach. International Quality of Life Assessment. J. Clin. Epidemiol..

[12] Beaton D.E., Bombardier C., Guillemin F., Ferraz M.B. (2000). Guidelines for the process of cross-cultural adaptation of self-report measures. Spine (Phila PA 1976).

[13] Wild D., Grove A., Martin M., Eremenco S., McElroy S., Verjee-Lorenz A., Erikson P., ISPOR Task Force for Translation and Cultural Adaptation (2005). Principles of Good Practice for the Translation and Cultural Adaptation Process for Patient-Reported Outcomes (PRO) Measures: Report of the ISPOR Task Force for Translation and Cultural Adaptation. Value Health.

[14] George D., Mallery P. (2003). SPSS for Windows Step by Step: A Simple Guide and Reference, 11.0 Update.

[15] Fleiss J.L. (1981). Statistical Methods for Rates and Proportions.

[16] Cohen J. (1988). Statistical Power Analysis for the Behavioral Sciences.

[17] Radosavljevic N., Lazovic M., Nikolic D., Petronic I., Radosavljevic Z., Jeremic A. (2012). Influence of selective comorbidity predictors on functional recovery after hip fracture in an older population. Biomed. Pap. Med. Fac. Univ. Palacky Olomouc Czech Repub..

[18] Piyaphanee N., Sirikuntaramas S., Sumboonnanonda A., Farhat W.A. (2016). Validity and Reliability of the Thai Version of Dysfunctional Voiding Symptom Score (DVSS) Questionnaire. J. Med. Assoc. Thai..

[19] McDonald R.P. (1981). The dimensionality of tests and items. Br. J. Math. Stat. Pshychol..

[20] Cronbach L.J. (1951). Coefficient alpha and the internal structure of tests. Psychometrika.

[21] Revelle W. (1979). Hierarchical cluster-analysis and the internal structure of tests. Multivar. Behav. Res..

[22] McDonald R.P. (1999). Test Theory: A Unified Treatment.

[23] Zinbarg R.E., Revelle W., Yovel I., Li W. (2005). Cronbach’s α, Revelle’s β, and McDonald’s ωH: Their relations with each other and two alternative conceptualizations of reliability. Psychometrika.

[24] Tang W., Cui Y., Babenko O. (2014). Internal Consistency: Do we really know what it is and how to assess it?. J. Psychol. Behav. Sci..

[25] Farver J.A.M., Kim Y.K., Lee-Shin Y. (2000). Within cultural differences: Examining individual differences in Korean American and European American preschoolers’ social pretend play. J. Cross. Cult. Psychol..

[26] Messick S. (1995). Validity of psychological assessment: Validation of inferences from persons’ responses and performances as scientific inquiry into score meaning. Am. Psychol..

[27] Strauss M.E., Smith G.T. (2009). Construct validity: Advances in theory and methodology. Annu. Rev. Clin. Psychol..

