# Supplementary material for: Validation of Serbian Version of Dysfunctional Voiding Symptom Score (DVSS) Questionnaire
Source: J Clin Med. 2018 Aug 14;7(8):217. doi: 10.3390/jcm7080217 (PMC6111336; doi:10.3390/jcm7080217)
Supplement: Supplementary file 1 [file jcm-07-00217-s001.pdf]

Table S1. Construct validity of the Serbian version of the Dysfunctional Voiding Symptom Score.

Test items	Construct validity (Gender) r value*	Construct validity (Education) r value*
Item 1	-0.082	-0.082
Item 2	0.000	0.000
Item 3	0.227	0.057
Item 4	0.065	-0.239
Item 5	0.157	0.220
Item 6	0.244	-0.035
Item 7	0.114	-0.228
Item 8	0.177	-0.177
Item 9	-0.266	-0.207
Item 10	0.000	-0.136

All correlations are not significant (p>0.05); *Pearson's correlation.

Table S2. Divergent validity of the Serbian version of the Dysfunctional Voiding Symptom Score.

Test items	Divergent validity (Gender) Male (mean±SD)	Female (mean±SD)	P value*	(Education) Preschool (mean±SD)	Primary school (mean±SD)	P value*
Item 1	1.84±0.75	1.72±0.74	0.570	1.84±0.80	1.72±0.68	0.570
Item 2	1.92±0.91	1.92±0.81	1.000	1.92±0.86	1.92±0.86	1.000
Item 3	1.52±0.65	1.84±0.75	0.113	1.64±0.64	1.72±0.80	0.696
Item 4	1.84±0.94	1.96±0.94	0.653	2.12±0.88	1.68±0.95	0.095
Item 5	1.48±0.77	1.68±0.48	0.275	1.44±0.71	1.72±0.54	0.124
Item 6	1.16±0.55	1.44±0.58	0.088	1.32±0.63	1.28±0.54	0.810
Item 7	2.08±0.64	2.24±0.78	0.431	2.32±0.69	2.00±0.71	0.112
Item 8	1.68±0.80	1.96±0.79	0.220	1.96±0.74	1.68±0.85	0.220
Item 9	2.16±0.69	1.80±0.65	0.062	2.12±0.73	1.84±0.62	0.150
Item 10	1.52±1.50	1.52±1.50	1.000	1.72±1.46	1.32±1.52	0.347

*Unpaired t-test.

Table S3. Explained variability between gender and education for each item of the Serbian version of the Dysfunctional Voiding Symptom Score.

Test items	(Gender) F value*	η²(%)	(Education) F value*	η²(%)
Item 1	0.327	0.68	0.327	0.68
Item 2	0.000	0	0.000	0
Item 3	2.603	5.14	0.155	0.32
Item 4	0.204	0.42	2.888	5.69
Item 5	1.220	2.48	2.450	4.86
Item 6	3.031	5.93	0.058	0.12
Item 7	0.630	1.29	2.621	5.18
Item 8	1.547	3.12	1.547	3.12

Item 9	3.640	7.05	2.138	4.26
Item 10	0.000	0	0.902	1.84
